# The human brain through the lens of somatic mosaicism

**DOI:** 10.3389/fnins.2023.1172469

**Published:** 2023-05-12

**Authors:** Sara Bizzotto

**Affiliations:** Sorbonne Université, Institut du Cerveau (Paris Brain Institute) ICM, Inserm, CNRS, Hôpital de la Pitié Salpêtrière, Paris, France

**Keywords:** somatic mosaicism, human brain, neurodevelopment, brain aging, neurodevelopmental disorders, neurodegeneration

## Abstract

Every cell in the human brain possesses a unique genome that is the product of the accumulation of somatic mutations starting from the first postzygotic cell division and continuing throughout life. Somatic mosaicism in the human brain has been the focus of several recent efforts that took advantage of key technological innovations to start elucidating brain development, aging and disease directly in human tissue. On one side, somatic mutation occurring in progenitor cells has been used as a natural barcoding system to address cell phylogenies of clone formation and cell segregation in the brain lineage. On the other side, analyses of mutation rates and patterns in the genome of brain cells have revealed mechanisms of brain aging and disorder predisposition. In addition to the study of somatic mosaicism in the normal human brain, the contribution of somatic mutation has been investigated in both developmental neuropsychiatric and neurodegenerative disorders. This review starts with a methodological perspective on the study of somatic mosaicism to then cover the most recent findings in brain development and aging, and ends with the role of somatic mutations in brain disease. Thus, this review underlies what we have learned and what is still possible to discover by looking at somatic mosaicism in the brain genome.

## Introduction

We have long believed that the human genome is the same in each cell of the body, and that rare somatic mutations occurring during life often due to the exposure to external agents such as smoke and UV light, are the cause of cancer. However, more recently, we have learned that the human body carries as many different genomes as the number of cells it is composed of, a phenomenon referred to as somatic mosaicism. This is due to the accumulation of somatic mutations (or variants) starting with the first postzygotic cell division and continuing during the whole life at rates and patterns that are specific to each tissue and cell type.

Somatic mutations, once occurred, permanently mark the genome. For this reason, the study of somatic mutation has proven effective at elucidating organism and tissue development but also disease predisposition and pathology insurgence. Several recent efforts have focused on somatic mutations in the human brain, revealing unique insights into development, aging and pathology. This review covers the latest findings in brain somatic mutation, and put them in perspective to underlie the unique insights somatic mutation is providing into the human brain, and the future potential of this field.

### Methods and technologies to study somatic mutation

**Figure 1:**
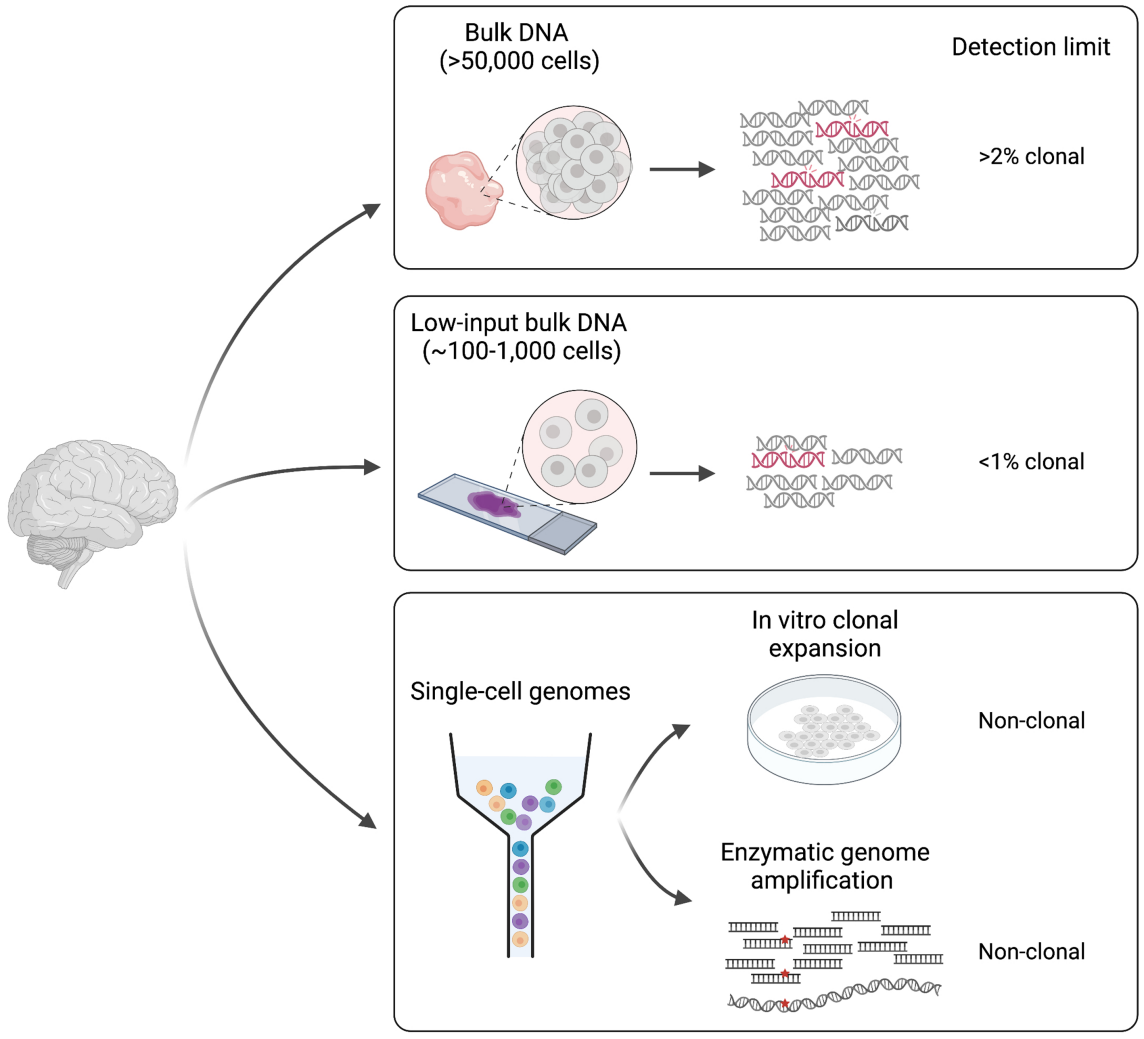
Different approaches for the study of somatic mutation. Methodologies can be divided in two main categories: (1) bulk DNA-sequencing (including low-input) and (2) single cell DNA-sequencing. Classic bulk DNA strategies use biopsies made of >50,000 cells and usually present a detection limit of >2% mosaicism (clonal). Low-input bulk methods start from as low as ~100–1,000 cells and have a lower detection limit of <1% clonal. Finally, single-cell technologies require genome amplification either through *in vitro* clonal expansion or through enzymatic genome amplification, and can detect non-clonal mutations present in single genomes. Figure created with BioRender.com.

The study of somatic mutation requires specific technologies and analytic tools. The choice of sample, the library preparation method and the sequencing technology must be adapted to the question(s) of interest. Indeed, different methodologies present specific detection limits that go from somatic mutations with >2% mosaicism in the sampled tissue to somatic mutations present in single-cell genomes (Figure 1).

Some of the studies conducted until now on the human brain have used deep (>200X) sequencing of bulk DNA extracted from biopsies made of thousands of cells (Baldassari et al., 2019a,b; Bizzotto et al., 2021; Fasching et al., 2021; Breuss et al., 2022). This approach allows detection of a wide range of variant allele frequencies (VAFs, calculated as the number of sequencing reads displaying the mutation over the total number of reads covering the mutant locus), and it is sensitive enough to detect somatic mutations present in >2% of the total cells in the biopsy (>1% VAF). However, the main limitation of this approach is the inability to detect extremely low (<1%) VAFs that represent rare mutations in the tissue, which might be relevant in polyclonal tissues such as the human brain, where many multiple progenitor cells contribute to a small tissue area. Bulk low-input sequencing technologies can overcome such limitation by sequencing DNA extracted from as few as 100–1,000 cells (Ellis et al., 2021). However, these technologies require at least few cycles of DNA amplification, which can introduce DNA polymerase errors that can be difficult to discriminate from true somatic events during analysis. Duplex consensus sequencing technologies solve this problem by using a library preparation strategy that allows the recognition of the two strands of the same DNA molecule after sequencing (Schmitt et al., 2012; Kennedy et al., 2014; Abascal et al., 2021). By these means, PCR errors are recognized as those that are present in copies of the same strand. Furthermore, sequencing artifacts are discriminated as those present in individual reads associated with one strand. Such solutions allow to detect rare, low VAF somatic mutations, and can provide good measures of somatic variant rates and abundance in human tissues (Abascal et al., 2021; Coorens et al., 2021).

Bulk sequencing methods are used to identify mutations that are shared by multiple cells in the tissue (clonal somatic mutations). Although this is useful for studies that are interested in mutations that may have a bigger impact at the global tissue level, it is not sensitive enough to address the cumulative effect of somatic mutations in non-replicating differentiated cells such as neurons in the postnatal brain. For this, whole-genome amplification (WGA) methods have been developed for detection of somatic mutations at the single-cell level (Spits et al., 2006; Bae et al., 2018; Fasching et al., 2021; Gonzalez-Pena et al., 2021; Xing et al., 2021; Luquette et al., 2022). The main WGA strategies applied until now in the brain include: (1) clonal expansion in culture (Bae et al., 2018) and (2) enzymatic genome amplification (Spits et al., 2006; Gonzalez-Pena et al., 2021; Xing et al., 2021). The former can be applied to proliferating cells or embryonic stem cells after nuclear transfer from any other cell type however, it is subjected to cell culture somatic artifacts often associated with hypoxic conditions. Several methods exist for enzymatic genome amplification that can be applied to any cell type (Spits et al., 2006; Gonzalez-Pena et al., 2021; Xing et al., 2021). The main problem associated with these technologies is the introduction of artifact variants during amplification that need to be filtered during data analysis. One limitation common to all single-genome sequencing approaches is that they are still difficult to apply to many multiple replicates. While clonal expansion in culture is experimentally elaborate, enzymatic methods still have elevated costs, which has limited existing studies to sample sizes of less than 200 single-genomes.

Although next-generation sequencing (NGS)-based methods are effective in the study of somatic single-nucleotide variants (sSNVs) and small (less than ~40 bp) insertion/deletions (indels), detecting somatic structural variants (SVs) such as large copy number variants (CNVs) in NGS data is still challenging and requires sophisticated computational solutions. For this, long-read third-generation sequencing methods represent a valid alternative. However, although studies have been successful in identifying germline SVs in long-read data, somatic SVs have not been explored enough yet. Thus, application of long-read sequencing to such purpose requires further investigation (Jenko Bizjan et al., 2020; Fujimoto et al., 2021).

In parallel to library preparation and sequencing methods, detection of somatic mutations requires computational tools specifically adapted to discriminate between low-frequency artifacts, germline mutations, and true somatic events. A plethora of pipelines are now available, and most of the studies privilege consensus between different methods applied to the same data. Recently, the Brain Somatic Mosaicism Network (BSMN) has published a consensus of best practices for calling sSNVs in brain bulk DNA deep sequencing data that has 65% genome-wide detection sensitivity, and relies on multiple filtering steps to get rid of library and sequencing artifacts and germline variants (Wang et al., 2021). In non-cancer studies, where somatic mutations that are shared with other tissues are still relevant (e.g., lineage tracing of human development), the challenge is that there is no matched control available to filter non-somatic germline mutations. Several callers have recently been introduced that address this issue by using features obtained from raw data, machine learning or image-based representations coupled with convolutional neural network (CNN)-based classification to detect somatic mutations with high sensitivity and precision in the absence of a matched control (Huang et al., 2017; Dou et al., 2020; Yang et al., 2023). Callers specifically designed to detect somatic mutations in single-cell WGS were also introduced that use either read phasing or leveraging of mutation signatures and allele balance to discriminate between true somatic mutations and artifacts introduced during WGA (Bohrson et al., 2019; Luquette et al., 2022).

Finally, a major challenge associated to somatic mosaicism remains the ability to identify somatic mutations in specific cell types populating the same tissue. This is especially relevant in the human brain, since it contains hundreds of different cell types that have specific developmental trajectories and properties. High-throughput single-cell transcriptomics and epigenomic data are now widely used to assign cell identities. However, genotyping of somatic mutations in such data is still limited by the sparse genome coverage given by these technologies (Bizzotto et al., 2021). Although identification of somatic mutations in specific cell types is now possible thanks to approaches such as cell-type sorting, G&T-seq (Genome and Transcriptome sequencing), PRDD-seq (Parallel RNA and DNA analysis after Deep-sequencing), GoT (Genotyping of Transcriptomes), and single-cell transcriptomics coupled with long-read sequencing, all these approaches still present several limitations (Macaulay et al., 2015; Nam et al., 2019; Huang et al., 2020; Bizzotto et al., 2021; Koboldt et al., 2021; Breuss et al., 2022; Townsend et al., 2023). While sorting is limited to broad and abundant cell types that can be targeted by specific known markers, G&T and PRDD-seq are still costly, time-consuming, low-throughput, or limited to targeted genes and genomic loci. GoT and long-read sequencing, on the other hand, have until now proven effective only for a subset of somatic mutations present in abundant transcripts. Thus, existing technologies are still very difficult to apply to low VAF somatic mutations and rare cell types, which underlies the need to develop more efficient high-throughput methods.

### Somatic mosaicism and human brain development

Since the first studies in 2015, somatic mutations have been shown to function as a tool to identify and track cellular clones in the human brain (Evrony et al., 2015; Lodato et al., 2015; Bizzotto and Walsh, 2022). The first study identified somatic endogenous retroelements in single-neurons and brain bulk DNA, and showed that spontaneous retrotransposition events that occur during human brain development can function as markers to analyze clone spreading in the cerebral cortex (Evrony et al., 2015). Retrotransposition events, however, are quite infrequent during development (<1 per terminally-differentiated neuronal genome), which limits their utility for the study of cell phylogenies (Evrony et al., 2012; Erwin et al., 2016). Indeed, more recent studies have focused on sSNVs that occur at a much higher frequency of at least 1–4 per genome per cell division (Bae et al., 2018; Ye et al., 2018; Rodin et al., 2021). sSNVs permanently label the genome of a cell and its descendance, except in the case of extremely rare events of somatic loss-of-heterozygosity. Furthermore, since the likelihood of the same rare mutation (excluding those frequent in the population) occurring twice in the same individual is extremely low, sSNVs act as unique markers. Thus, being frequent, unique, permanent and cumulative, sSNVs possess all the properties needed by a reliable lineage tracing tool. Since sSNVs label virtually every cell division of human brain development, they are ideal for studying cell phylogenies.

**Figure 2:**
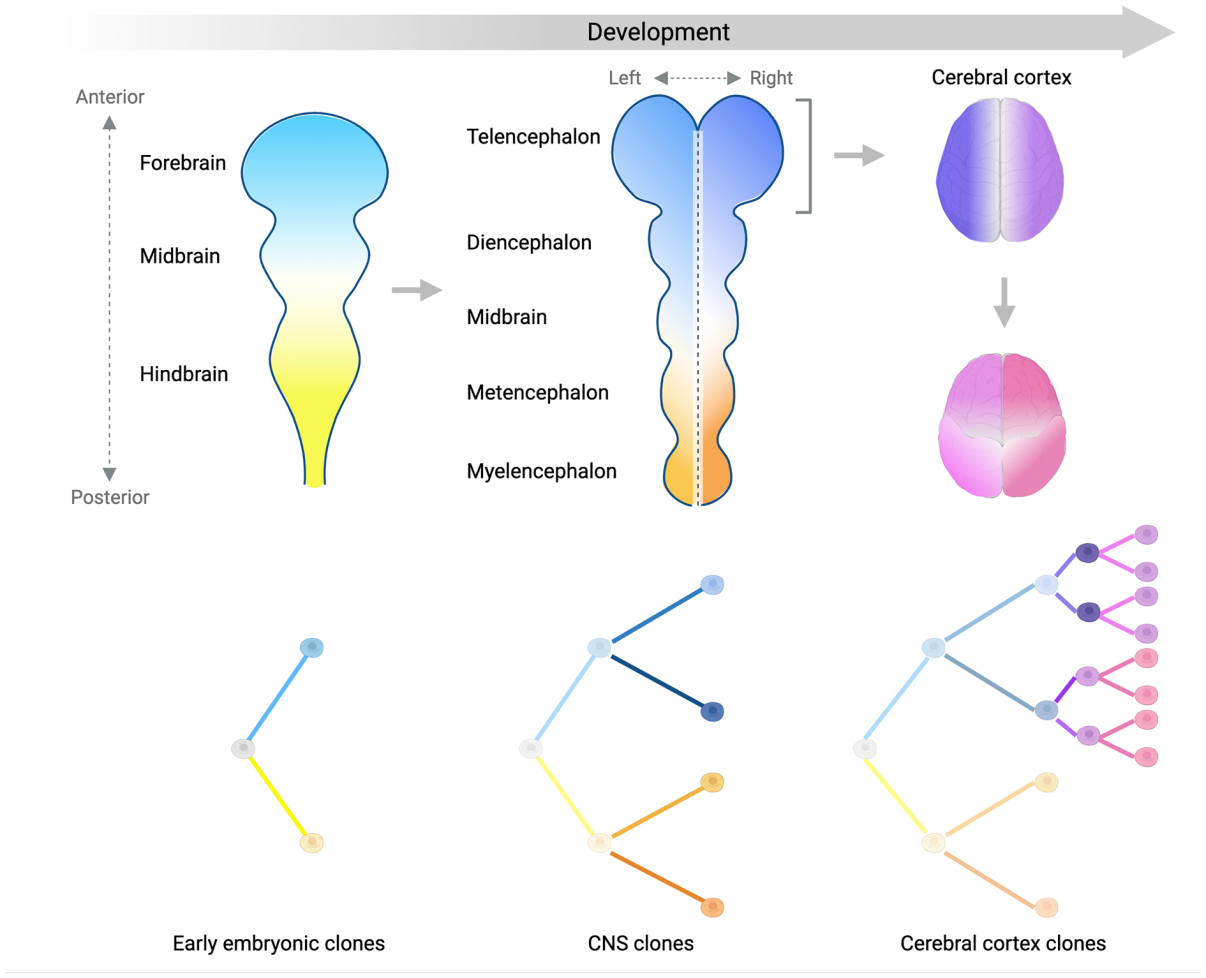
Lineage segregation during brain development. Schematic summarizing what we have learned so far about brain development from analyses of somatic mutation distribution patterns. Color gradients display the spatial distribution of separate (sub)-lineages in the developing central nervous system (CNS) over time. Trees in the bottom panels show the relationship between different (sub)-lineages. The antero-posterior axis is established first at early stages of embryonic development (left panel) and is represented by two lineages giving rise mostly to the anterior (light blue) or the posterior (light yellow) parts of the CNS primordium. As the CNS develops into more sub-regions, the left–right axis starts being established by sub-lineages of the blue and yellow clones (middle panel). Focusing on the forebrain (right panels), the left–right axis separating the two hemispheres (dark blue and purple) is established before the separation between the frontal lobe and more posterior lobes of the cortex (magenta and pink sub-lineages). Later on, the ventral-dorsal axis is established (not shown). Figure created with BioRender.com.

Studies conducted so far have managed to identify several clones and map their contributions to different brain regions and non-brain tissues, and have used this information to place in a temporal order some landmark steps of human brain development (Figure 2). We showed that ~50–100 founder progenitors of the human forebrain are produced by lineages that originate before gastrulation from the first cell divisions of the human embryo (Bizzotto et al., 2021; Bizzotto and Walsh, 2022). Another study suggested that brain lineage founder cells may segregate according to the antero-posterior axis of the central nervous system (CNS) primordium (formed by forebrain, midbrain and hindbrain) at very early stages of embryonic development (Fasching et al., 2021). The forebrain subsequently gives rise to the most anterior telencephalon and the diencephalon, while the hindbrain splits into metencephalon and myelencephalon. Analysis of clonal sSNVs showed that the forebrain and cerebellar primordia (metencephalon), likely split before the formation of the midline that separates the left and right hemispheres of the forebrain (Breuss et al., 2022), which means that the left–right axis is established after the specification of the neural tube into the most anterior forebrain and the most posterior hindbrain. Finally, within the forebrain, a first clone diffusion barrier seems to be established along the midline, while along the anterior–posterior axis clones may be able to diffuse more freely until later stages (Coorens et al., 2021; Breuss et al., 2022) (Figure 2). Around 90–200 progenitors were estimated at the time of forebrain lateralization, consistent with the number of forebrain founder progenitors mentioned above (Breuss et al., 2022). sSNV distribution along the antero-posterior axis of the cortex showed that the frontal lobe and more posterior lobes constitute two broadly definable lineage clusters (Figure 2), probably due to the presence of the central sulcus and the Sylvian fissure, which may constitute a physical diffusion barrier limiting clonal spreading across regions (Bizzotto et al., 2021; Breuss et al., 2022). Finally, analyses of somatic mutations in sorted populations of glial cells (astrocytes, oligodendrocytes, and microglia) and excitatory and inhibitory neurons strongly suggested that ventral and dorsal regions separate after the formation of the left–right and anterior–posterior axes (Breuss et al., 2022). Despite these very interesting findings regarding the order in which different domains and axes are formed, our knowledge about segregation of progenitor cells in the brain lineage is still limited, and will require further, more in depth studies of somatic mutations across brain regions.

The human cerebral cortex is composed of ~170 billion cells and more than a 100 different cell types classified so far. This cellular complexity is reached through a huge number of cell divisions that involve different types of progenitor cells. For this reason, the clonal structure of the cortex is hard to decipher but somatic mutations are offering some interesting insights. Analyses of geographical distribution of clonal sSNVs in the cortex strongly suggested that younger clones arising later during development and presenting lower mosaicism distribute over narrower regions (Lodato et al., 2015; Bizzotto et al., 2021; Breuss et al., 2022). This observation suggests that each cortical region is a patchwork of cells that belong to independent intermingling clones. Although it has been difficult so far to reconstruct cell divisions of committed neural progenitors in the cortex due to the extremely low VAF (<1%) of somatic mutations that mark such divisions, few studies have managed to address how earlier, easier to identify, embryonic cell divisions contribute to the cortex. We and others showed that lineages generated in the early embryo contribute asymmetrically on average to the cortex, with a high inter-individual variability that suggests that individual clones may undergo differential expansions and/or bottleneck events (Bizzotto et al., 2021; Coorens et al., 2021; Fasching et al., 2021; Breuss et al., 2022).

The mammalian cerebral cortex contains two broad cell type categories that are neuronal and non-neuronal glial cells, and we and others showed that in humans these two classes of cells are generated in different percentages from early pre-neurogenesis embryonic cell divisions (Bizzotto et al., 2021; Breuss et al., 2022). If we split these two main categories further, we find several cell types that are produced by distinct progenitor niches and follow characteristic migratory patterns. In the mouse, excitatory neurons, astrocytes and some oligodendrocytes are born from dorsal ventricular zone progenitors, while inhibitory neurons and other oligodendrocytes are derived from ventrally located progenitors. Somatic mutations characterized until now in different cell type populations seem to confirm this general pattern also in humans (Huang et al., 2020; Breuss et al., 2022). However, we know from a recent study that in humans a small proportion of inhibitory neurons might share dorsal cortical progenitors with excitatory neurons (Delgado et al., 2022), though this has not been confirmed yet by lineage tracing studies using somatic mutations in human tissue.

The cerebral cortex is a laminated structure composed of six identifiable layers that form in an inside-out manner. PRDD-seq studies managed to identify this inside-out order of formation of cortical layers in humans, showing at the same time that 10 or more excitatory neuron progenitors contribute to a specific cortical radial column. The same study also showed that medial, lateral and caudal ganglionic eminence (MGE, LGE, and CGE) interneurons are generated over the same developmental time window, except maybe for a proportion of CGE-derived LAMP5+ interneurons, and especially those co-expressing SST, that seem to be generated later. Similar to the mouse, PVALB+ and SST+ MGE-derived interneurons were enriched in infragranular cortical layers IV to VI and II to VI, respectively, while CGE-derived LAMP5+ and VIP+ interneurons tended to occupy upper layers (Huang et al., 2020). However, the study found no evidence for an inside-out pattern characterizing interneuron development, which is still debated in the mouse as well (Ang et al., 2003; Rudy et al., 2011; Huang et al., 2020). Future studies will need to characterize later more cell-type-restricted somatic mutations in order to reveal the finer properties of cell type production and distribution in the cortex.

### The aging human brain

The process of aging is accompanied by an increase in the predisposition to certain disorders such as neurodegeneration and cancer. In the aging brain, somatic mutations accumulate linearly in a process called *genosenium* (Lodato et al., 2018). The most recent estimates show that somatic mutations in aging human neurons accumulate at rates of ~16–17 sSNVs and 2–3 indels per genome per year such that while right after birth each neuron contains ~100–200 sSNVs and ~10–30 indels, at more advanced ages (>70 years old), sSNVs are on the order of ~1,000–2,000, and indels on the order of ~250–350 (Abascal et al., 2021; Luquette et al., 2022). Very recent data additionally characterized somatic mutations in aging oligodendrocytes, showing that these are surprisingly different from neurons and accumulate sSNVs 69% faster (~27 per genome per year) and indels 42% slower (~1.8 per genome per year), probably due to different mechanisms of mutation (see below). Thus, oligodendrocytes in the brain of an 80 years old individual carry an order of 2,000–3,000 sSNVs and 150–200 indels, significantly different from the burden observed in neurons (Ganz et al., 2023).

**Figure 3:**
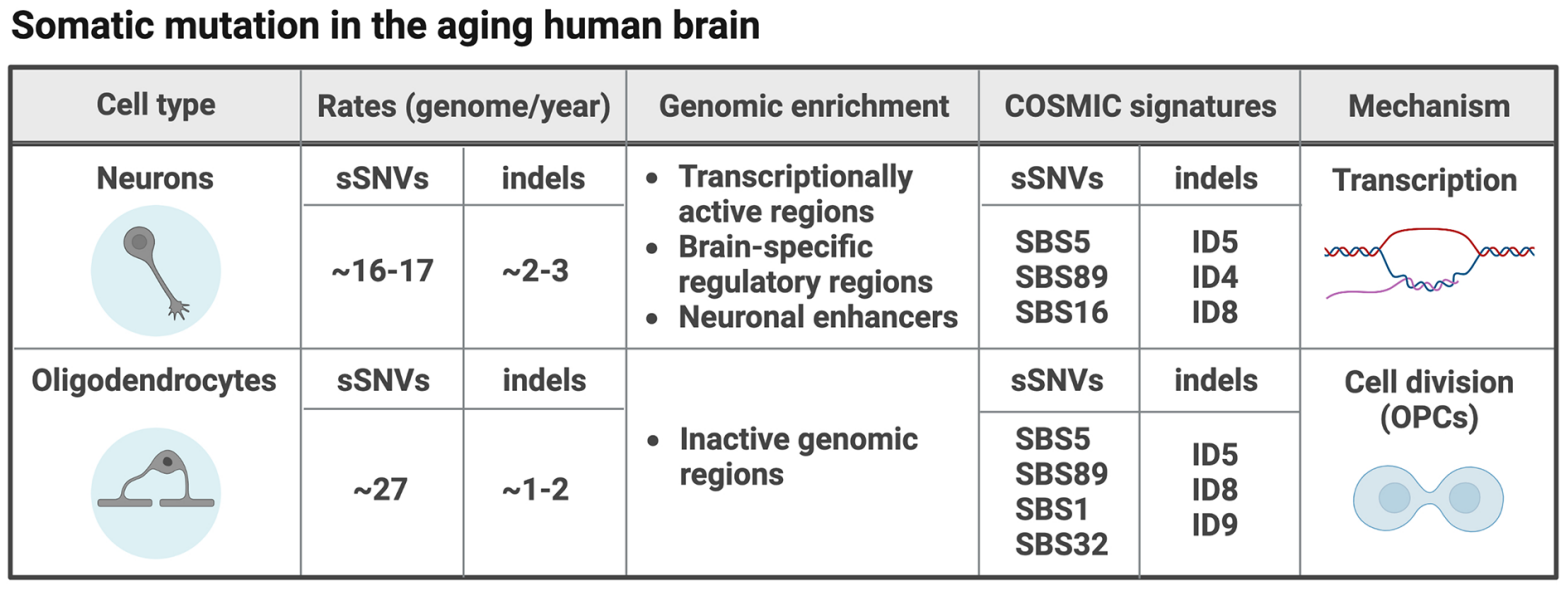
Somatic mutation in different cell types in the aging human brain. The table summarizes what we have learned so far about somatic mutation in two different cell types (neurons and oligodendrocytes) in the aging human brain (Ganz et al., 2023). Figure created with BioRender.com.

The mechanisms of somatic mutation in the aging brain are not completely understood and vary between cell types. Recent studies have analyzed the genomic distribution of somatic mutations compared to several genomic co-variates (e.g., transcriptional activity, chromatin accessibility, replication timing, etc). Furthermore, mutation characteristics summarized in what are called signatures, were used to dissect mutation mechanisms. Signatures are spectra of somatic mutations obtained by the type of substitution and the trinucleotide context in the case of sSNVs, and the size, nucleotides affected and presence on repetitive and/or microhomology regions in the case of indels. Signatures are extracted and decomposed from a set of somatic mutations identified in tissue or single-cell genomes and can be fitted to signatures identified in many types of cancer as reported in the Catalogue Of Somatic Mutations In Cancer (COSMIC) (Tate et al., 2019). Such analyses have provided a way to understand the processes underlying somatic mutation in the brain. Analyses of genomic distribution of sSNVs and indels in aging neurons showed that somatic mutation seems to be driven mostly by transcriptional activity. Indeed, both sSNVs and indels were enriched in transcriptionally active functional regions and brain-specific regulatory regions (Lodato et al., 2018; Luquette et al., 2022; Ganz et al., 2023). Furthermore, both sSNVs and indels show highly significant enrichment in neuronal enhancers (Luquette et al., 2022). Mutational signatures identified in neurons resemble COSMIC single base substitution (SBS)5 and SBS89, and indel ID5 and ID8 (Luquette et al., 2022; Ganz et al., 2023). SBS5, ID5 and ID8 are clock-like signatures that accumulate with age independently of cell division, while SBS89 has no clear etiology. Consistent with the idea of neuronal somatic mutation being driven by transcription, the transcription-associated signatures SBS16 and ID4 were also found in aging neurons (Ganz et al., 2023). Recently, we found that oligodendrocytes accumulate mutations due to strikingly different mechanisms compared to neurons in the same brains (summarized in Figure 3). In oligodendrocytes, cell division seems to play an important role, consistent with the replenishment of oligodendrocytes by their precursor cells (OPCs) during the whole postnatal life. Oligodendrocyte sSNVs and indels are overall enriched in inactive genomic regions and although oligodendrocytes display accumulation of signatures also found in neurons such as SBS5, SBS89, ID5 and ID8, signatures specific to oligodendrocytes were identified such as the cell division signature SBS1 and SBS32, and signature ID9, usually found in a large fraction of gliomas and other brain tumors. Coherent with this, oligodendrocyte mutation density profiles across the genome correlated with those of glial-derived tumors (Ganz et al., 2023). Despite the association of neuronal somatic mutation with transcription levels, there is no data so far supporting the enrichment of somatic mutations in specific genes or gene networks and pathways. However, in our recent study we tested specifically cancer-associated genes in oligodendrocytes compared to neurons, and found that oligodendrocyte but not neuronal sSNVs were biased towards cancer-associated genes and even more towards genes mutated in CNS tumors, with the top tumors being oligodendroglioma and pilocytic astrocytoma (Ganz et al., 2023).

Accumulation of somatic mutations in aging brain cells informs on cell-type-specific disease predisposition. As we will see more in depth in the next section, somatic mutation in neurons is linked to neurodegeneration (Miller et al., 2022). In glial cells, however, somatic mutation may play a role in predisposing to tumor insurgence as we become older. A study investigating the burden of clonal somatic mutations in normal human brains found no correlation with age (Ganz et al., 2021). However, another study found that the proportion of brains carrying >100 sSNVs (hypermutable brains) raised with age (Bae et al., 2022). Hypermutability seems to be due to expansions of single or very few clones due to driver mutations that hitchhike all other mutations belonging to the same lineage. Indeed, hypermutable brains were enriched for damaging mutations in genes implicated in cancer (Bae et al., 2022). Since there is very little neuronal turnover in the postnatal brain, such clonal expansions are either congenital or the product of postnatal expansions within the glia lineage. Indeed, an increase in clonal oncogenic somatic mutations was observed in the white matter of the normal human cerebral cortex compared to the adjacent grey matter (Ganz et al., 2021). Despite these observations, contrary to other tissues (Genovese et al., 2014; Martincorena et al., 2015, 2018; Lee-Six et al., 2019; Yokoyama et al., 2019; Moore et al., 2020), oncogenic mutations in the human brain do not seem to increase with age (Ganz et al., 2021), maybe reflecting the fact that brain oncogenic clonal expansions over time can easily result in disease, thus eliminating the individuals carrying such clonal expansions from the control cohorts. It seems however important to underlie that studies conducted until now focused on relatively high VAFs, thus the increase with age of micro-clones carrying oncogenic mutations cannot be excluded at this time.

### Somatic mosaicism in neuropsychiatric and neurodegenerative disorders

Current estimates attribute 5–10% of the missing genetic heritability of more than 100 human disorders to somatic mutations (Yang et al., 2020). Somatic mutations are a known cause of, or have been implicated in several brain disorders from developmental neuropsychiatric disorders such as focal epilepsy, autism spectrum disorders (ASD) and schizophrenia (SCZ) to neurodegeneration and Alzheimer’s disease (AD) (Lim et al., 2015; D’Gama et al., 2017; Lim et al., 2017; Winawer et al., 2018; Baldassari et al., 2019a,b; Rodin et al., 2021; Sherman et al., 2021; Maury et al., 2022; Miller et al., 2022).

In the human genome, exons and areas of open chromatin are particularly vulnerable to somatic mutation during development (Rodin et al., 2021). Pathogenic somatic mutations in the mechanistic target of rapamycin (mTOR) pathway genes can cause focal cortical dysplasia (FCD) spectrum disorders that are associated with pharmaco-resistant epilepsy, such as FCD Type 2, hemimegalencephaly (HME) and tuberous sclerosis complex (TSC). Mutations identified so far cause hyperactivation of the mTOR pathway either through monoallelic gain-of-function (GoF) of an mTOR activator (*AKT3*, *PIK3CA*, *RHEB*, and *MTOR*), or through bi-allelic loss-of-function (LoF) of a repressor (*TSC1/2*, *NPRL2/3*, and *DEPDC5*) often due to a germline mutation followed by a somatic mutation in the second allele of the same gene (Poduri et al., 2012; Lim et al., 2015; D’Gama et al., 2017; Lim et al., 2017; Baldassari et al., 2019a,b; Lee et al., 2021). The mTOR pathway is a main regulator of cell growth and proliferation and indeed its hyperactivation causes hallmarks of the disease such as cortical dyslamination and presence of dysmorphic cells of abnormal size in the brain tissue (Blumcke et al., 2021). FCD mutations are often not found in patients’ blood and are thus thought to occur in the brain lineage during cortical development. Although it has been shown that the size of the lesion correlates with the percentage of mutant cells, and that bigger lesions often involve both neurons and glial cells (D’Gama et al., 2017; Baldassari et al., 2019a,b), the exact time in development when FCD somatic mutations occur remains to be elucidated. Few studies have suggested that the excitatory neuron lineage, and especially outer radial glial cells (oRG) in the dorsal forebrain might be majorly affected in FCD (Pollen et al., 2019; Andrews et al., 2020; Chung et al., 2023) however, the exact cell types involved in the disorder pathophysiology remain unclear. Furthermore, even if a clear link between mTOR pathway genes and FCD spectrum disorders has been established, ~50–60% of cases have no clear genetic etiology. Recently, other genes and pathways have been implicated in focal brain malformations, such as genes involved in protein glycosylation (*SLC35A2*) or genes regulating synaptic function and calcium dynamics (Winawer et al., 2018; Bonduelle et al., 2021).

FCD lesions are often found in extra-temporal locations in the cortex, with FCD type 2 preferentially affecting the frontal lobe (Kabat and Król, 2012) however, mesial temporal lobe epilepsy (MTLE), which is the most common focal epilepsy subtype, originates in the hippocampus (Lamberink et al., 2020). A very recent study addressed somatic mosaicism in this pathology by deep whole-exome sequencing (WES), and found mutations increasing the activity of the Ras/Raf/MAPK pathway in genes such as *PTPN11*, *KRAS*, *SOS1*, *BRAF*, and *NF1*. Such mutations were found in the hippocampus of lesional cases with visible mesial temporal sclerosis (MTS) but were undetectable in the adjacent temporal cortex and in non-lesional MTLEs. Low VAFs (0.8–3.3%) were found in cases without additional lesions such as low-grade epilepsy-associated tumors or FCD, suggesting later developmental occurrence (Khoshkhoo et al., 2022). These findings introduce the possibility of a pathway-specific susceptibility characterizing different brain regions, with the hippocampus being affected by mutations in the Ras/Raf/MAPK pathway and the frontal cortex by mutations in the PI3K/Akt/mTOR pathway. Although this remains purely speculative, we could hypothesize that this pathway-specific susceptibility has to do with the differential transcriptional activity of the two pathways in different brain regions during development or at early postnatal stages.

While developmental focal epilepsies are often monogenic disorders and thus their genetic etiology easier to investigate, the genetics underlying other developmental neuropsychiatric disorders such as ASD and SCZ is more difficult to elucidate due to their complex multigenic nature. Recent studies have shown a contribution of somatic mutations to these disorders (Dou et al., 2017; Krupp et al., 2017; Rodin et al., 2021; Sherman et al., 2021). Analyses of brain deep WGS data revealed an excess of mosaic sSNVs in ASD compared to neurotypical individuals in critical brain enhancers associated with genes specifically expressed in the brain (Rodin et al., 2021). A separate study also showed a significant contribution of large (>4 Mb) CNVs to ASD risk in 0.2% of probands (Sherman et al., 2021). Somatic mutation contribution to SCZ has been explored through deep WGS of purified neuronal populations, which identified two mechanisms contributing specifically to SCZ compared to control neurons, referred to as “*skiagenesis*” (Maury et al., 2022). A significant increase in sSNVs proximal (+/−1 Kb) to the midpoint of active transcription factor binding sites (TFBS) was observed, with enrichment seemingly related to fetal development but not brain-specific. Indeed, sSNV enrichment was seen at sites for many individual transcription factors (TFs) essential for early embryonic, craniofacial, and nervous system development. Signature analyses suggested a first mechanism generating these mutations as a consequence of prenatal oxidative damage that does not get effectively repaired due to TF binding that hinders the DNA repair machinery. A second mechanism was revealed by a 61.8-fold enrichment of CpG > GpG in active TFBS at promoters compared to the expected C > G genome-wide rate. C > G and C > A transversions at CpG sites reflect a footprint of enzymatic demethylation involving the creation of an abasic site through resection of oxidated methyl-cytosine. TF binding may similarly obstruct the repair of abasic sites before replication, thus leading to CpG transversions (Maury et al., 2022).

While developmental neuropsychiatric disorders and/or disorder risk are both associated with somatic mutations shared by multiple cells (clonal mutations), non-clonal somatic mutations accumulating in aging neurons have been linked to neurodegenerative disorders such as Cockayne Syndrome (CS), Xeroderma Pigmentosum (XP) and AD (Lodato et al., 2018; Miller et al., 2022). CP and XP are caused by defects in DNA damage repair (DDR), and are associated with neurodegeneration and premature aging. Single neuron genomes from post-mortem brains of CS and XP individuals showed ~2.3-fold and ~2.5-fold excess of sSNV compared to age-matched neurotypical neurons, respectively (Lodato et al., 2018). A higher burden of sSNV accumulation during aging was also observed in PFC and hippocampal AD neurons compared to neurotypical controls. A signature showing a pronounced increase in AD compared to control included C > A substitutions associated with oxidative damage to guanine nucleotides and transcription-coupled nucleotide excision repair (TC-NER) deficiency (Jager et al., 2019; Kucab et al., 2019; Alexandrov et al., 2020; Miller et al., 2022). Thus, sSNVs in AD seem to increase due to the accumulation of high levels of inflammation-associated reactive oxygen species (ROS) that overwhelm the TC-NER. Although the exact mechanisms remain to be fully clarified, these studies highlight a clear link that appears to exist between increased burden of somatic mutation in neuronal genomes and neurodegeneration.

## Discussion

Somatic mutations in the human brain genome provide a record of each cell’s history that we have started deciphering. While mutations occurring in progenitor cells offer a retrospective forensic lineage tree that describes cell divisions and clone formation (Bizzotto et al., 2021), post-mitotic non-clonal mutations represent a linear timer of the cell’s life (Ganz et al., 2023). Recent studies have highlighted how different brain cell types accumulate somatic mutations with specific rates and patterns (Ganz et al., 2023). Further elucidating cell-type-specific rates of somatic mutation will greatly improve our understanding of human brain development but also postnatal cell phylogenies (e.g., glial cell replenishment or postnatal neurogenesis) by combining the unfolding of cell divisions with a temporal dimension of when any two cells split from a common ancestor (Ganz et al., 2023).

We now know that somatic mutations are linked to certain developmental brain pathologies such as FCD, ASD, and SCZ (D’Gama et al., 2017; Baldassari et al., 2019a,b; Rodin et al., 2021; Sherman et al., 2021; Maury et al., 2022). How somatic mutations impact development and brain function in these pathologies remains for the most part unclear. This is due to the limited knowledge of the normal developmental process of the human brain. Elucidating such aspects with further basic studies on the normal building of the clonal architecture of the human brain, including also cell type-specific patterns, will certainly contribute to clarify pathology insurgence. In addition to the developmental consequences of pathogenic mutations, the underlying causes of somatic mutation in these pathologies remain to be explored. Future studies will need to address this question. The same is true for the consequences of mosaic pathogenic mutations compared to their germline counterpart, and how different levels of mosaicism can impact brain function for distinct mutant genes and mutation types.

Recent studies have shown how certain pathological states are associated to increased somatic mutation rates in the human brain and to disease-specific mechanisms (Lodato et al., 2018; Miller et al., 2022). Although current knowledge seems to suggest that increased somatic mutation in AD is due to oxidative damage due to the disorder, the exact role of increased rates of somatic mutation in neurodegeneration remains unclear, as well as the limit beyond which somatic mutations are not tolerated, thus leading to cell death.

The speed at which new technologies are introduced in the genetics field promises to boost many future studies that will contribute to further decipher human cells and tissues by dissecting the genome at unprecedent resolution. Somatic mutations provide a way of studying directly the human brain starting from available tissue. These studies are nicely complementing what we learn from animal and *in vitro* models by providing information on human brain development, aging, and pathology that is not accessible otherwise.

## Author contributions

The author confirms being the sole contributor of this work and has approved it for publication.

## Funding

SB is supported by the Horizon2020 Research and Innovation Program Marie Skłodowska-Curie Actions (MSCA) Individual Fellowship (grant agreement no. 101026484—CODICES).

## Conflict of interest

The author declares that the research was conducted in the absence of any commercial or financial relationships that could be construed as a potential conflict of interest.

## Publisher’s note

All claims expressed in this article are solely those of the authors and do not necessarily represent those of their affiliated organizations, or those of the publisher, the editors and the reviewers. Any product that may be evaluated in this article, or claim that may be made by its manufacturer, is not guaranteed or endorsed by the publisher.
